# Primary Renal Lymphoma Identified in a Robot-Assisted Laparoscopic Nephroureterectomy Specimen

**DOI:** 10.1089/cren.2016.0054

**Published:** 2016-05-01

**Authors:** Jacob Jipp, Daniel Sadowski, Paul Kay, Bradley Schwartz

**Affiliations:** ^1^ University of Iowa Carver College of Medicine, Iowa City, Iowa.; ^2^ Division of Urology, Southern Illinois University School of Medicine, Springfield, Illinois.; ^3^ Department of Pathology, HSHS St. John's Hospital, Springfield, Illinois.

## Abstract

***Background:*** Although renal involvement is often present in non-Hodgkin's lymphoma (NHL), primary renal NHL is a rare diagnosis.

***Case Presentation:*** We present a case report of a 72-year-old asymptomatic male who underwent a robot-assisted laparoscopic radical nephroureterectomy on an atrophic left kidney with evidence of an infiltrating mass. Pathology report demonstrated a grade 1 follicular lymphoma.

***Conclusion:*** Lymphoma is a differential that should be considered when evaluating a renal mass. Chemotherapy and radiation are the mainstays of treatment.

## Introduction and Background

Postmortem analysis of patients with non-Hodgkin's lymphoma (NHL) has shown that renal involvement is quite common. However, primary renal NHL is not as frequently seen and analysis of it has mostly been limited to case reports and case series. These patients often present with symptoms consistent with other primary malignancies of the upper tract, including flank pain, renal failure, or hematuria.

In this case report, we present an asymptomatic gentlemen who was found to have a left renal mass upon imaging for a recurrent incisional hernia. Grade 1 follicular lymphoma was diagnosed from pathology analysis, and further work-up postoperatively revealed stage IV disease, including involvement of bone marrow on biopsy and lymph nodes on positron emission tomography (PET) scan. We defined primary renal NHL by the patient's lymphoma being clinically dominant in the kidney, even in the presence of disseminated disease.^1^

## Presentation of Case

A 72-year-old white male with extensive smoking history presented to our clinic by referral from his general surgeon. We were requested to further evaluate an atrophic left kidney and infiltrating left renal mass. In 1962, he had a splenectomy secondary to trauma that required midline and left subcostal incisions. In the year before our consultation, he had undergone three separate abdominal surgeries: laparoscopic hiatal hernia repair, incisional hernia repair, and recurrent incisional hernia repair with mesh. He reported a persistent bulge in the upper aspect of his incisional hernia repair.

**FIG. 1.**
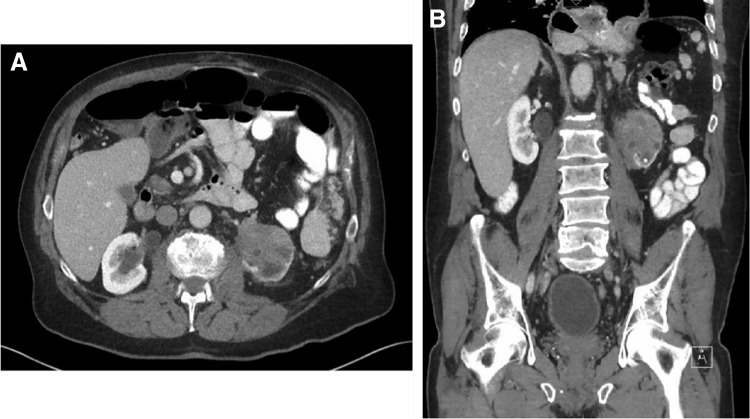
CT scan with axial **(A)** and coronal **(B)** views showing an infiltrating mass in an atrophic left kidney. CT, computed tomography.

He underwent CT imaging as part of the work-up to evaluate for a recurrent incisional hernia, which showed marked left renal atrophy and a 5.5 cm infiltrative left lower pole mass causing hydronephrosis (Fig. 1). Management options were discussed with the patient, and he wished to forego a renal biopsy and proceed with surgical removal. We performed a robot-assisted laparoscopic radical nephroureterectomy with excision of bladder cuff. All dissections were performed robotically, including the distal ureter and bladder cuff. General surgery performed a laparoscopic repair of his incisional hernia with mesh in conjunction with our surgery.

**FIG. 2.**
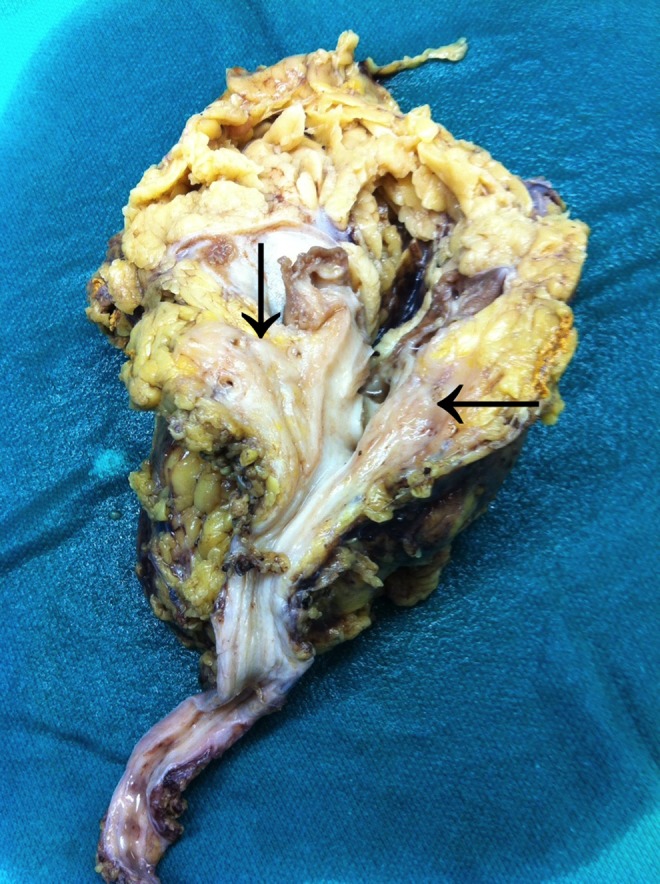
Gross nephroureterectomy specimen with follicular lymphoma (*arrows*) appearing as a tan-colored mass.

Gross analysis of the nephroureterectomy specimen showed an 8 × 4.5 × 4 cm kidney. Bisection of the kidney showed a dense infiltrative process involving the soft tissue of the hilum as well as the fat of the renal sinus (Fig. 2). The mass was noted to extensively involve the specimen and measured 9 × 5 × 3.5 cm. It extended close to the inked surface of the kidney and also traveled along vessels to the vascular margins.

**FIG. 3.**
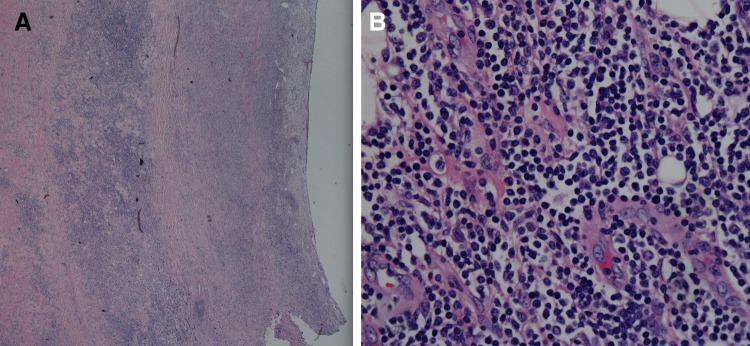
Renal pelvis with infiltrating follicular lymphoma invading into the submucosal connective tissue **(A)** and high-power view (400×) of extensive infiltration of lymphocytes (*blue*) within the renal pelvis connective tissue **(B)**.

**FIG. 4.**
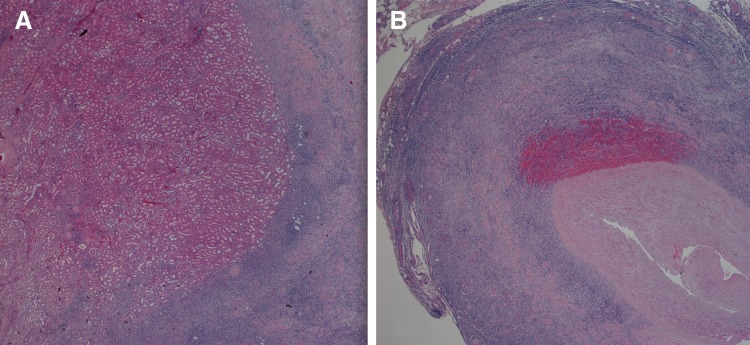
Invasion of follicular lymphoma into the renal parenchyma **(A)** and distal ureter **(B)**.

Microscopic analysis of the 9 cm renal mass depicted a monomorphic lymphoid process (Figs. 3 and 4). Immunohistochemistry was performed at our local hospital that demonstrated the lymphoid cells were CD20 and CD79 positive, while lacking staining for CD3, CD5, CD10, and CD68. The specimen was sent to Mayo Clinic in Rochester, MN, for further evaluation. Their pathologist noted a diffuse infiltrate of small lymphocytes in a sclerotic background. Fluorescence *in situ* hybridization analysis of the specimen indicated that 100% of the cells had a BCL2 separation as well as a BCL6 separation. The diagnosis was follicular lymphoma, grade I (of III).

The patient recovered well postoperatively. His cystogram showed no extravasation and his foley catheter was removed. He was seen by oncology for further management of his lymphoma. Bone marrow biopsy demonstrated 5% involvement of the bone marrow by the follicular lymphoma. PET scan showed evidence of lymphadenopathy in the retrocrural region adjacent to his hiatal hernia and in the right lower pelvic lymph nodes. There were no signs of any visceral metastases. Given the bone marrow involvement, the final diagnosis was stage IVA, grade 1 follicular lymphoma. His beta-2 microglobulin level was elevated, which portends a poorer prognosis. He began chemotherapy with rituximab (monoclonal antibody that binds B-lymphocyte CD20) and bendamustine (alkylating agent). He has finished two cycles of treatment to date.

## Discussion and Literature Review

In this case report, we presented an individual with primary renal follicular lymphoma. Research on these patients is lacking since the disease is so rare; most of the literature consists of case reports and case series. Regardless, our patient's age was consistent with the typical presentation of middle-aged to elderly adults. Ferry and colleagues reported unilateral disease to be more common than bilateral disease, which also fits with the clinical picture of our patient.^2^

Primary renal lymphoma was a diagnosis that was frequently brought into question until the early 1990s. The understanding that renal involvement of NHL commonly existed, but the absence of lymphoid tissue in the kidneys brought doubt to primary lymphomas arising in the renal system. Although research has continued to exponentially grow, there is no clear understanding of the pathogenesis of primary renal lymphoma. Multiple hypotheses have been developed to try to explain how an organ devoid of lymphoid tissue can be the primary site for lymphomatous disease, but most of these hypotheses were developed many years ago. One article that examined nearly 1500 cases of extranodal NHL hypothesized that a pathologic proliferative response of local lymphoid is the precursor to the disease.^3^ Others believed that an inflammatory process recruits lymphoid cells to the area, and although those cells are in the extranodal area, the necessary mutation occurs that begets the oncologic process.^4^ In terms of renal-specific disease, the renal capsule has an abundance of lymphatics, which may allow the neoplastic cells to penetrate the parenchyma and develop the disease.

## Conclusion

Evidence in the literature supports the notion that CT-guided fine needle biopsy is the preferable means to obtain a tissue diagnosis. Once the diagnosis is confirmed, management with chemotherapeutic agents and involved-field radiation therapy is the mainstay of treatment. However, these lymphomas can often mimic other renal neoplasms and no tissue had previously been obtained to confirm diagnosis. We opted for surgical management to remove this infiltrating mass in an atrophic kidney and established a diagnosis of follicular lymphoma. The patient was then referred to oncology for further management of disseminated disease.

## Abbreviations Used

CT: computed tomography
PET: positron emission tomography
NHL: non-Hodgkin's lymphoma
